# Abdominal fascia dehiscence: is there a connection to a special microbial spectrum?

**DOI:** 10.1007/s10029-022-02679-7

**Published:** 2022-09-22

**Authors:** P. V. Stropnicky, F. Kandemir, M. Schäffer, J. Pochhammer

**Affiliations:** 1 Clinic for General, Visceral, Thoracic, Paediatric and Transplant Surgery, University Hospital Schleswig-Holstein, Arnold-Hellerstr. 3 Campus Kiel, 24105 Kiel, Germany; 2 Department of Visceral, General, and Thoracic Surgery, Marienhospital Stuttgart, Stuttgart, Germany; 3 Clinic for Trauma Surgery & Orthopedics, Klinik am Eichert (Alb-Fils-Kliniken GmbH), Göppingen, Germany

**Keywords:** Surgical site infection, Wound infection, Antimicrobial management, Microbial pathogens, Fascial dehiscence, Burst abdomen, Enterococci

## Abstract

**Introduction:**

Acute fascia dehiscence (FD) is a threatening complication occurring in 0.4–3.5% of cases after abdominal surgery. Prolonged hospital stay, increased mortality and increased rate of incisional hernias could be following consequences. Several risk factors are controversially discussed. Even though surgical infection is a known, indisputable risk factor, it is still not proven if a special spectrum of pathogens is responsible. In this study, we investigated if a specific spectrum of microbial pathogens is associated with FD.

**Methods:**

We performed a retrospective matched pair analysis of 53 consecutive patients with an FD after abdominal surgery in 2010–2016. Matching criteria were gender, age, primary procedure and surgeon. The primary endpoint was the frequency of pathogens detected intraoperatively, the secondary endpoint was the occurrence of risk factors in patients with (FD) and without (nFD) FD.

**Results:**

Intraabdominal pathogens were detected more often in the FD group (*p* = 0.039), with a higher number of Gram-positive pathogens. Enterococci were the most common pathogen (*p* = 0.002), not covered in 73% (FD group) compared to 22% (nFD group) by the given antibiotic therapy. Multivariable analysis showed detection of Gram-positive pathogens, detection of enterococci in primary laparotomy beside chronic lung disease, surgical site infections and continuous steroid therapy as independent risk factors.

**Conclusion:**

Risk factors are factors that reduce wound healing or increase intra-abdominal pressure. Furthermore detection of Gram-positive pathogens especially enterococci was detected as an independent risk factor and its empirical coverage could be advantageous for high-risk patients.

## Introduction

Acute fascia dehiscence (FD) describes the separation of the sutured abdominal wall immediately or in the postoperative course after abdominal surgery. Cohort studies report incidences of 0.4–3.5% of FD after abdominal surgery [1, 2]. FD has to be treated by a second laparotomy and has serious consequences for the patient. Increased mortality rates of up to 45% and a reduced 5-year-survival rate after FD are described [2–4]. Prolonged hospitalization, with a possible intensive care stay, the development of further complications such as pneumonia, delirium or re-operations due to recurrences can be the consequence [5–7]. In addition, incisional hernias were found in up to 70% of cases within 10 years after FD treatment [3, 8–10].

Regardless of the consequences for the individual patient, FD is a considerable, avoidable financial burden for the health care system due to prolonged hospital stays, longer intensive therapy and the necessary reoperation with longer convalescence time of the patient [6, 9, 11].

For a long time, it was assumed that the surgical material and the suturing technique were the cause or a relevant risk factor for FD [2, 12]. Despite innovations in perioperative treatment and the development of antibacterial and elastic suture material, the incidence of FD has not been significantly reduced. Therefore, patient's own risk factors are considered to have a relevant influence [13, 14].

This has led to various retrospective studies being conducted to identify risk factors, some of which have produced contradictory results [1, 5, 6, 9, 11, 15, 16]. Further studies were conducted to develop a risk model for preoperative identification of increased risk, although these risk models have not yet been established in clinical practice [11, 16].

However, most studies agree that surgical site infections are associated with a significant risk of developing FD [6, 7, 15]. Gram-positive pathogens have been found more frequently in wound infections after caesarean section, and a connection with FD is suspected [17]. *Enterococcus faecalis* is also known to cause slow destruction of heart valves with a high rate of surgical revisions despite its low virulence. Because of its intrinsic resistance, extensive and prolonged antibiotic therapy is necessary, but it is not practical as standard therapy after abdominal surgery, leaving one with a clinical dilemma [18]. Whether a specific pathogen or a specific spectrum of microorganisms is causative for FD and what influence the chosen antibiotic therapy has on the development of FD has not yet been investigated.

Therefore, in this study, we investigated whether specific bacterial contamination is associated with the occurrence of FD and how this is changed by postoperative antibiotic therapy to potentially determine the need for extended perioperative antibiotic prophylaxis in high-risk patients.

## Methods

We performed a retrospective matched pair analysis of all patients with FD after laparotomy or after laparoscopically assisted procedures (primary laparotomy; PL) in Marienhospital Stuttgart. Fifty-three patients were found and matched to 53 patients without FD (FD vs. nFD) in 2010–2016. Matching criteria were gender, age group, primary procedure and surgeon. Diagnosis of FD was made by clinical appearance and secondary laparotomy (SL) was performed on the day of suspected FD. In all cases, fascial closure was performed continuously using sling of strength 0 PDS Plus (Johnson&Johnson^®^, New Brunswick, USA). All patients received preoperative antibiotic prophylaxis which was chosen according to the indication and type of procedure performed and our clinical internal standard.

Primary endpoint was the frequency of microbial pathogens which were found in the abdominal swabs in PL. Secondary endpoint was the occurrence of known risk factors in FD. Therefore, we reviewed the surgery, anesthesia and discharge reports, inpatient documentation and laboratory and microbiological findings. Intraabdominal swabs and microbiological examinations of PL and SL were collected and compared. A second-look surgery was performed in eight and seven patients, respectively. The swab of the second-look procedure was included in the pathogen spectrum of PL. The antibiotic therapy was noted and compared to microbiological examinations to examine if the antibiotic therapy was effective.

The statistical analysis was done with the statistical program IBM^®^ SPSS Statistics^®^ version 24.0 (IBM^®^ Corp., New York, USA). For a normal distribution of values, the *t* test for independent samples was used, in all other cases *U* test according to Mann–Whitney. Nominally scaled influencing factors were tested for their possible influence on the development of FD using Pearson's chi-square test or exact Fisher test. For all nominally scaled dichotomous variables, the odds ratio with the corresponding 95% confidence interval was also given for a better representation of a possible correlation.

Multivariable analysis was performed for development of FD, with all factors in Tables 2 and 3 significant in univariate analysis, and consisted of multiple regression analysis with backward elimination. The procedure was rerun until no explanatory variable was left that could be removed without markedly worsening the prediction of the endpoint. Chronic lung disease, surgical site infections, detection of Gram-positive pathogens in PL, detection of enterococci in PL and continuous steroid therapy remained in the final model. For all tests, significant differences were defined for a *p* value < 0.05.

All patients consent to the processing of their data upon admission to hospital and their data were processed anonymously. The hospital's internal ethics committee approved our study.

## Results

During our study period, 53 patients with FD were matched (*n* = 53). FD occurred on a median of postoperative day 8. The earliest occurrence was day 2 and the latest was day 25. FD was most common after colorectal surgery (47.2%), followed by jejunum and ileum surgery (18.9%), stomach and duodenum surgery (17%), liver/bile and pancreas surgery (7.5% each) and abdominal wall surgery (1.9%). The primary surgery was one of the matching criteria (Table 1).

**Table 1:** Matching criteria

	FD (*n* = 53)	nFD (*n* = 53)	*p* value
Sex			
Female	23 (43.4)	23 (43.4)	1.0
Male	30 (56.6)	30 (56.6)	1.0
Age difference	0 ± 4.88		0.993
Type of primary surgery			
Colon/Rectum	25 (47.2)	25 (47,2)	1.0
Jejunum/Ileum	10 (18.9)	10 (18.9)	
Stomach/Duodenum	9 (17)	9 (17)	
Liver/Biliary	4 (7.5)	4 (7.5)	
Pancreas	4 (7.5)	4 (7.5)	
Abdominal wall	1 (1.9)	1 (1.9)	
Surgeon			
A	11 (20.8)	12 (22.6)	0.98
B	5 (9.4)	5 (9.4)	
C	15 (28.3)	15 (28.3)	
D	4 (7.5)	5 (9.4)	
E	10 (18.9)	10 (18.9)	
F	8 (15.1)	6 (11.8)	

*FD* fascia dehiscence group, *nFD* no fascia dehiscence group

Data are reported as absolute numbers (percentage), *p* values are calculated using the Pearson’s chi-squared

Table 2 shows the patient-dependent and pre-existing risk factors for FD. Long-term steroid therapy, preoperative anemia or hypalbuminemia, and previous laparotomy < 6 months ago were found significantly more often in the FD group.

**Table 2:** Risk factors

	FD (*n* = 53)	nFD (*n* = 53)	OR (95% CI)	Univariate *p* value	Multivariate *p* value
Smoking	12 (24.5)	7 (13.7)	2.0 (0.7–5.7)	n.s	–
Arterial hypertension	39 (73.6)	37 (69.8)	1.2 (0.5–2.8)	n.s	–
Diabetes Mellitus	12 (22.6)	13 (24.5)	0.9 (0.4–2.2)	n.s	–
Chronic lung disease	14 (26.4)	7 (13.2)	2.4 (0.9–6.4)	0.088	< 0.01
Continuous steroid therapy	16 (30.2)	6 (11.3)	3.4 (1.2–9.5)	0.017	0.02
Malignant primary disease	32 (60.4)	27 (50.9)	1.5 (0.7–3.2)	n.s	–
Chemotherapy within last 30d	4 (7.5)	0	0.5 (0.4–0.6)	0.041	n.s
Chemotherapy within last 6 M	12 (22.6)	7 (13.2)	1.9 (0.7–5.4)	n.s	–
Radiation	5 (9.4)	4 (7.5)	1.3 (0.3–5.0)	n.s	–
Laparotomy within the last 30 days	10 (18.9)	3 (5.7)	3.9 (1.0–15.0)	0.038	n.s
Laparotomy within the last 6 months	16 (30.2)	5 (9.4)	4.2 (1.0–12.0)	0.007	n.s
Abdominal pre-surgery	41 (77.4)	39 (73.6)	1.2 (0.5–3.0)	n.s	–
Hypoalbuminemia	33 (62.3)	20 (37.7)	2.7 (1.2–6.0)	0.012	n.s
Anemia (pre-existent)	47 (88.7)	38 (71.7)	3.1 (1.1–8.7)	0.028	n.s
Relaparotomy*	7 (13.2)	8 (15.1)	0.9 (0.3–2.6)	n.s	–
Anastomosis insufficiency	6 (11.3)	2 (3.8)	3.3 (0.6–16.9)	n.s	–
Pneumonia	15 (28.3)	7 (13.2)	2.6 (1.0–7.0)	0.055	n.s
Cough	7 (13.2)	1 (1.9)	7.9 (0.9–66.8)	0.027	n.s
Vomiting	14 (26.4)	7 (13.2)	2.4 (0.9–6.4)	0.088	n.s
Ascites	13 (24.5)	5 (9.4)	3.1 (1.0–9.5)	0.038	n.s
Wound infection	41 (77.4)	19 (35.8)	6.1 (2.6–14.4)	< 0.001	< 0.01

*FD* fascia dehiscence group, *nFD* no fascia dehiscence group, *OR* Odds Ratio with a 95% confidence interval (CI), *n.s.* not significant)

*Programmed relaparotomy/second look procedure or unplanned relaparotomy between primary and secondary laparotomy

Data are reported as absolute numbers (percentage); *p* values are calculated using the Pearson’s chi-squared or Fisher’s exact test

The frequency of an emergency indication did not differ between the groups (41.5 vs. 37.7%, *p* = n.s.). The approach was mostly a median laparotomy (88.7 vs. 79.2%, *p* = n.s.). The mean duration of surgery was 2 h in FD and 2.5 h in nFD (*p* = n.s.). Intraoperative evidence of peritonitis in PL was found more often in the FD group (57.7 vs 39.6%, *p* = 0.068).

Anastomotic insufficiency has occurred in six cases of FD and in two cases of nFD (*p* = 0.27). In five out of six affected patients in the FD group, this complication was first discovered during SL.

Conditions related to an increased abdominal pressure in the postoperative course occurred more often in the FD group. Postoperative pneumonia showed a trend but did not differ significantly (*p* = 0.055).

Multivariable analysis retained chronic lung disease, surgical site infections and continuous steroid therapy in the final model. *P* values were < 0.01 for the first factors and 0.02 for steroid use, which means that all five are independent risk factors (Table 2).

Intraoperative smears of PL were taken in 60% and 62% in FD and nFD cases, respectively. In the FD group, smear pathogens were found more frequently (*p* = 0.039), especially Gram-positive pathogens (*p* = 0.008). In detail, *Enterococcus species* (*p* = 0.002), *Clostridium species* (*p* = 0.024) and *Candida species* (*p* = 0.034) were found in the FD group more frequently.

In the multivariate analysis, detection of Gram-positive pathogens in PL and detection of enterococci in PL were included in the final model and showed to be independent risk factors with a *p* value of < 0.01 (Table 3).

**Table 3:** Microbiological analysis in PL

	FD* (*n* = 32)	nFD* (*n* = 33)	OR (95% CI)	Univariate *p* value	Multivariate *p* value
Smear with germ evidence	26 (81.2)	19 (57.6)	3.2 (1.0–9.8)	0.039	
Detection of Gram-positive pathogens	23 (71.9)	13 (39.4)	3.9 (1.4–11.1)	0.008	< 0.01
Detection of Gram-negative pathogens	17 (53.1)	16 (48.5)	1.2 (0.5–3.2)	n.s	–
Enterococcus	19 (59.4)	7 (21.2)	5.4 (1.8–16.2)	0.002	< 0.01
*Enterococcus faecalis*	11 (34.4)	2 (6.1)	8.1 (1.6–40.4)	0.004	n.s
*Enterococcus faecium*	7 (21.9)	4 (12.1)	2.0 (0.5–7.8)	n.s	–
*Enterococcus species*	2 (6.3)	1 (3.0)	2.1 (0.2–24.8)	n.s	–
*Escheria coli*	7 (21.9)	8 (24.2)	0.9 (0.28–2.8)	n.s	–
*Enterobacter species*	6 (18.8)	5 (15.2)	1.3 (0.35–4.7)	n.s	–
*Klebsiella species*	5 (15.6)	2 (6.1)	2.9 (0.5–16.0)	n.s	–
*Bacteroides species*	5 (15.6)	10 (30.3)	0.4 (0.1–1.4)	n.s	–
*Clostridium species*	5 (15.6)	0	0.5 (0.3–0.6)	0,024	n.s
*Pseudomonas species*	3 (9.4)	1 (3.0)	3.3 (0.3–33.6)	n.s	
*Staphylococcus aureus*	4 (12.5)	2 (6.1)	2.2 (0.4–13.0)	n.s	
Coagulase-negative Staphylococci	1 (3.1)	3 (9.1)	0.32 (0.03–3.3)	n.s	
*Streptococcus species*	0	3 (9.1)	0.5 (0.4–0.6)	n.s	
*Candida species*	8 (25)	2 (6.1)	5.2 (1.0–26.6)	0.034	n.s
Other (*Prevotella species *etc*.*)	4 (12.5)	2 (6.1)	2.2 (0.4–13.0)	n.s	–

*FD* fascia dehiscence group, *nFD* no fascia dehiscence group, *OR* Odds Ratio with a 95% confidence interval (CI), *n.s.* not significant)

*With existing germ smear

Data are reported as absolute numbers (percentage); *p* values are calculated using the Pearson’s chi-squared or Fisher’s exact test

In SL, intraoperative smears were taken in 93% of patients. In comparison to PL, the proportion of enterococci, streptococci and coagulase-negative staphylococci increased, while the detection of enterococci showed the strongest increase in SL. *Clostridium species* were not found anymore (Table 4).

**Table 4:** Microbiological analysis of FD in primary laparotomy (PL) compared to in secondary laparotomy (SL)

	PL* (*n* = 32)	SL* (*n* = 49)
Smear with germ evidence	26 (81.2)	44 (89.8)
Gram-positive pathogens	23 (71.9)	42 (85.7)
Gram-negative pathogens	17 (53.1)	11 (22.4)
Enterococcus	19 (59.4)	33 (67.3)
*Enterococcus faecalis*	11 (34.4)	20 (40.8)
*Enterococcus faecium*	7 (21.9)	16 (32.7)
*Enterococcus species*	2 (6.3)	1 (2)
*Escheria coli*	7 (21.9)	7 (14.3)
*Enterobacter species*	6 (18.8)	5 (10.2)
*Klebsiella species*	5 (15.6)	1 (2)
*Bacteroides species*	5 (15.6)	4 (8.2)
*Clostridium species*	5 (15.6)	0
*Pseudomonas species*	3 (9.4)	3 (6.1)
*Staphylococcus aureus*	4 (12.5)	6 (12.2)
Coagulase-negative Staphylococci	1 (3.1)	4 (8.2)
*Streptococcus species*	0	3 (6.1)
*Candida species*	8 (25)	8 (16.3)
Other (*Prevotella species* etc.)	4 (12.5)	5 (10.2)

*PL* Primary Laparotomy, *SL* Secondary Laparotomy

Data are reported as absolute numbers (percentage)

*With existing germ smear

Forty-one patients (77.4%) in the FD group and 32 patients (60.4%) in the nFD group received a continued antibiotic therapy after PL. A cephalosporin with metronidazole was in the FD and nFD group in 50.9% and 37.7% of cases followed by a penicillin with a beta-lactamase inhibitor in 13.2% and 15.1% of cases and a carbapenem in 7.5% and 3.8% of cases the most used substances. The comparison of the postoperative antibiotic therapy to the microbiological examinations showed an incomplete coverage of pathogens in 80% and 40% of cases, respectively. This was mostly due to the fact that enterococci were not covered in 73% and 22% of cases. *Candida species* were not treated in 22% and 10% of cases (Table 5).

**Table 5:** Evaluation of postoperative anti-infective therapy

	FD (*n* = 41)*	nFD (*n* = 32)*	*p* value
Non-effective therapy	33 (80.5)	13 (40.6)	< 0.01
Enterococcus gap	30 (73.2)	7 (21.9)	< 0.01
Candida gap	9 (22.0)	3 (9.4)	n.s
Resistant germs (MRGN)	8 (19.5)	4 (12.5)	n.s

*FD* fascia dehiscence group, *nFD* no fascia dehiscence group, *n.s.* not significant, *MRGN* multi-resistant gram-negative bacteria

*Number of patients with postoperative continued anti-infective therapy

Data are reported as absolute numbers (percentage); *p* values are calculated using the Pearson’s chi-squared or Fisher’s exact test

## Discussion

FD is a serious complication in the postoperative course with serious consequences; therefore, it is of high priority to reduce the occurrence of FD [11]. Our study assessed the bacterial spectrum in patients developing FD compared to a matched control cohort. We found Gram-positive pathogens, especially enterococci, were significantly more often detected in the FD group and were shown to be an independent risk factor in the multivariate analysis.

Many patient-own, non-influenceable risk factors as well as influenceable risk factors in the context of surgery and postoperative treatment are discussed [1, 2, 4, 6, 9]. In the literature, advanced age and male gender are associated with a higher risk [2, 5, 11, 15]. Therefore, we included both as our matching criteria. Important risk factors were shown to be factors that lead to reduced healing, such as anemia or hypalbuminemia. Both lead to a lower supply of tissue, reduced oxygenation and reduced wound healing [2, 5, 6, 15]. Factors such as continued steroid use and chemotherapy within the last 30 days were also shown to be a risk as they generally affect cell proliferation and thus negatively influence healing [19–25]. However, in our study, radio- or chemotherapy performed more than 30 days ago was not a risk factor. We explain this by the fact that the effect of chemotherapy is no longer persistent and cell regeneration is, therefore, not compromised. This corresponds to the recommendation to pause chemotherapy for 4 weeks before and after surgery [20, 24].

In our study, both chronic lung disease and steroid therapy were shown to be independent risk factors in the multivariate analysis, which have also been described in the literature before. Steroid therapy is known to reduce wound healing [6, 11, 15].

A chronic cough could lead to an increase in abdominal pressure and, therefore, an increased risk of FD, in line with the literature, although in our study a cough alone was not shown to be an independent risk factor. Other known risk factors with abdominal pressure increase are ascites and vomiting [7, 9, 11, 16]. In our study, a clear connection between sudden increase in pressure in the course of coughing and rupture of the wound was found in two cases. In both cases, a ruptured abdomen was diagnosed immediately afterwards. In clinical practice, this should lead to avoiding an increase in intra-abdominal pressure. This could be prevented, for example, by the adequate administration of antiemetics, the placement of an ascitic drain or the implementation of respiratory therapy to prevent coughing.

Previous abdominal surgery more than 6 months ago was not shown to be a risk factor, while abdominal surgery within the last 6 months was associated with increased risk. This could be explained by the fact that repeated opening and subsequent closure of the abdomen leads to increased risk and fragility of the fascia. Another reason could be that the patients who had abdominal surgery within the last 6 months also had other risk factors, such as anemia, continued steroid use or recent chemotherapy in the context of a previous disease which was responsible for the previous surgery. This explanation would be strengthened by the fact that relaparotomy within the last 6 months was not shown to be an independent risk factor in the multivariate analysis. Van Ramshorst et al. and Samartsev et al. found no association between previous laparotomy and FD. However, the temporal distance to the relaparotomy was not specified [9, 11].

The performed access was not shown to be a risk factor in our case. This is in accordance with the literature [4, 6, 10, 13, 14]. Similarly, the surgery time was not shown to be a risk factor. This can be explained by the fact that our average surgery time was 2–2.5 h and thus too short to be recorded as an independent risk factor. In a study by Webster et al., surgery time > 2.5 h was shown to be a risk factor [16]. Other studies found no association between duration of surgery and FD [6, 9].

Contrary to the literature, the emergency indication was not shown to be a risk factor in our case [5, 11]. We explain this by the fact that it is not the emergency but the underlying disease that carries the increased risk. Therefore, an emergency surgery with intra-abdominal infection carries a higher risk than an emergency surgery without infection. In addition, a peritonitis already diagnosed in PL is associated with an increased risk in our study. In a study by Aksamija et al., of 44 patients with FD, 12 had emergency surgery. Aksamija et al. argue that patients who require emergency surgery are in poorer general health overall, i.e. have other risk factors, such as malnutrition, anemia or hypalbuminemia. Furthermore, the risk of contamination of the surgical area is greater and the surgeon's concentration at night may be affected. In this study, however, 16 patients already showed signs of peritonitis and 61% showed signs of infection [5].

The most undisputed risk factor of a FD in the literature and in our study is an infection, regardless of whether it occurs as a surgical site infection (SSI) or as an anastomotic insufficiency with peritonitis [1, 2, 5, 6, 9, 11, 15]. The presence of microbial pathogens leads to activation of the immune system with increased immigration of neutrophil leukocytes, release of tissue-degrading proteins, such as matrix metalloproteinases, inhibition of fibroblasts and thus lower collagen production. Endotoxins released by bacteria lead to the activation of collagenases and further collagen degradation. This results in poor wound healing and low tissue strength so that the risk of suture bursting is significantly increased when the intra-abdominal pressure is raised [11].

We found a dominance of Gram-positive pathogens compared to the literature, which showed to be an independent risk factor in the multivariate analysis. In the literature, Gram-negative bacteria such as *Klebsiella species* or *Escheria coli* are described as frequent pathogens of a SSI [26–28]. The difference to our study may be due to the fact that most patients in our study received antibiotic therapy that adequately covered these pathogens.

The detection of enterococci was particularly noteworthy. Enterococci were the most frequently detected pathogens and significantly more frequent in the FD group and showed to be an independent risk factor in the multivariate analysis.

An interesting fact is that *Enterococcus faecalis* is thought to be able to bind and activate human fibrinolytic protease plasminogen, leading to increased collagen degradation [29]. Slow destruction of the valves is already known from enterococcal endocarditis [18]. A similar process is conceivable with an infection of the fascia.

Furthermore, in 73% of the FD group, enterococci were the reason why antibiotic therapy was classified as not appropriate for the resistogram (Table 5). This is due to the fact that either a penicillin with beta-lactamase inhibitor or a cephalosporin such as cefotaxime and metronidazole was administered as PAP or as calculated therapy. Enterococci show intrinsic resistance to cephalosporins, beta lactam antibiotic substances are also often ineffective [27, 30]. Furthermore, there was an increase in the detection of enterococci in the SL. On one hand, this may be due to the fact that more of the patients in the SL than in the PL had a smear. On the other hand, the initial therapy was often not effective against enterococci and the adjustment was only possible after completion of the culture and resistance testing, which takes about 3–4 days, so that the increase in SL could also be explained by a selection through the given antibiotic therapy.

Whether enterococci are causative for FD cannot be proven in this study, so that extended antibiotic therapy with e.g. vancomycin does not make sense for all patients with abdominal surgery. However, their empirical coverage could be advantageous in high-risk patients and should be analyzed in further studies.

Also notable is the detection of candida. In 22% of the FD group, candida was not covered by the postoperative anti-infective therapy. This is due to the fact that in most cases the initial therapy does not contain an antimycotic. In the SL, the detection of candida showed a moderate decrease which may be due to the addition of an antimycotic after the result of the PL. In the multivariate analysis, the detection of *Candida species* was not found to be an independent risk factor, so its significance cannot be assessed in this study and could be a starting point for further studies.

## Limitations

The well-known disadvantages of a retrospective analysis apply to our study. The results depend on the quality of the data. Influencing factors, such as heterogeneity of the groups, cannot be excluded. The results of our studies must be interpreted considering the small number of cases of 53 patients in each groups, so that causal relationships cannot be definitely proven and confounders cannot be excluded.

The patients were matched after the initial procedures. We did not perform a stratification and evaluation of the smears with respect to the operated organ, as we did not consider this to be reliable due to the very small number of cases. Therefore, a different spectrum of microorganisms with respect to the operated organ is possible and should be considered in the initial antibiotic therapy. Further surveys, e.g. within the context of national registers, are necessary to also include different causes of peritonitis.

## Conclusion

In our study, we confirmed known factors that reduce wound healing or increase intra-abdominal pressure to increase the risk for FD. Especially SSI, chronic lung disease and chronic steroid use strongly correlated with occurrence of FD. Furthermore, in our study, we identified the detection of Gram-positive pathogens, especially enterococci in primary laparotomy as independent risk factors in multivariable analysis. Their empirical coverage could be advantageous in patients with high risk for FD.
